# Tuning Alignment, Strength, and Toughness in Functional Cellulose:Helux Filaments: A Molecular Trade-Off

**DOI:** 10.1021/acs.biomac.5c00128

**Published:** 2025-06-28

**Authors:** Saeed Davoodi, Faridah Namata, Tomas Rosén, Stephan V. Roth, Michael Malkoch, L. Daniel Söderberg, Fredrik Lundell

**Affiliations:** † Department of Engineering Mechanics, 7655 KTH Royal Institute of Technology, Stockholm 100 44, Sweden; ‡ Wallenberg Wood Science Center, KTH Royal Institute of Technology, Stockholm 100 44, Sweden; § Department of Fibre and Polymer Technology, KTH Royal Institute of Technology, Stockholm 100 44, Sweden; ∥ 28332 Deutsche Elektronen-Synchrotron DESY, Hamburg D-22607, Germany

## Abstract

The complex architecture of wood motivates studies of bioinspired materials that combine strength, toughness, and mechanical integrity. We explore the interplay between nanofiber alignment and molecular interactions in composite filaments formed from cellulose nanofibers (CNFs) and a dendritic polyampholyte, Helux. Helux enhances strength by 60% and increases toughness 5-fold through ionic bonding and thermal covalent cross-linking. However, wide-angle X-ray scattering (WAXS) reveals reduced nanofiber alignment in Helux-containing samples, resulting in a 25% decrease in stiffness—highlighting a trade-off between structural order and cohesion. Polarized optical microscopy (POM) and in situ small-angle X-ray scattering (SAXS) attribute this reduced alignment to enhanced rotary diffusion, driven by carboxylate groups of the Helux. With Helux, multivalent links across the nanofibers give a denser and tougher network with fewer voids. This behavior resembles lignin and hemicellulose interactions in wood, where flexibility and cohesion govern the performance.

## Graphical abstract

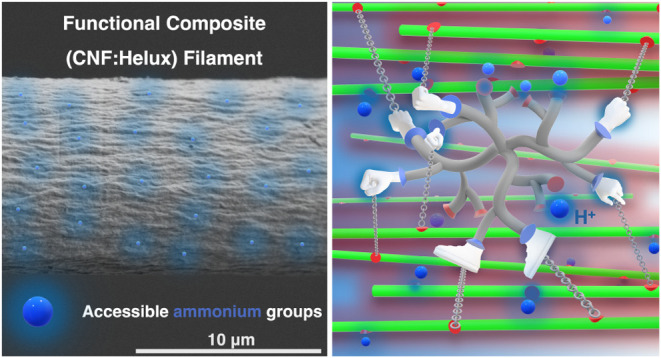

## Introduction

Nature has always served as an inspiration for scientists to design new materials with distinctive mechanical, optical, biological, and electrical properties. Numerous studies have been conducted to investigate the underlying mechanisms in biological materials and mimic their hierarchical architecture in order to make lightweight engineering materials for applications ranging from medicine to energy.^1^ Over the years, a conviction that there is potential in using wood, an abundant polymeric biomaterial, has developed. Like other plant-based materials, wood is a composite of polysaccharides building blocks; cellulose fibers within a lignin–hemicellulose matrix.^2^ The secondary cell wall layers of wood (especially S2, the thickest layer) exhibit anisotropic high stiffness and strength due to (1) alignment of semicrystalline, high-aspect-ratio, strong, and stiff cellulose nanofibers (CNFs) and (2) noncovalent interactions with an amorphous lignin-hemicellulose matrix.^3,4^ Cellulose chains interact via hydrogen bonding and van der Waals forces, forming a rigid network,^5^ while hemicellulose bridges between cellulose fibrils via hydrogen bonding, and lignin reinforces the structure through hydrophobic interactions.^6^ Translation of the extraordinary properties of CNFs into macroscale structures can be obtained by extracting these building blocks and minimizing nanoscale defects via controlled assembly, leading to strong networks transferring stresses from larger scales to the nanoscale.^2,7^ Microfluidic assembly is a technique that has been widely implemented to fabricate robust filaments with aligned biopolymer nanofibers.^8,9^ It is endowed that with the ability to optimize processing parameters and control in situ the physicochemical conditions, such as pH, salt, velocity gradients, etc., controlled self-assembly can be induced.^10^

Thanks to their potential applications and unique properties, composite filaments, where the nanofibers are complemented with an additive, have gained considerable attention. By incorporating biofunctional spider silk protein^11^ or either single-wall carbon nanotubes^12^ or PEDOT:PSS,^13^ filaments with biological function and conductivity can be prepared, respectively. Composite filaments show great potential, but the coupling between additives, processing, processability, and final properties is yet to be elucidated.

In this work, we study the processing and properties of CNF:Helux functional filaments. Helux, a dendritic polyampholyte with a pH-controllable net charge (Figure 1a),^14^ not only grants functionality to the cellulosic filament but also enhances mechanical performance. Previously, an in-depth study has been carried out on functional hybrid hydrogels derived from anionically charged high-aspect-ratio CNFs and Helux, which have provided insight into their microscale assembly mechanisms, colloidal stability, and mechanical and functional properties.^15^ At alkaline conditions (pH 10), the small Helux macromolecule can mix with anionic CNFs without disrupting the dispersed state at the nanoscale. CNF:Helux composite dispersions show thixotropic behavior and can form physical and reversibly cross-linked hydrogels at acidic conditions (pH 2),^15^ making it appropriate for double flow-focusing spinning.

**Figure 1:**
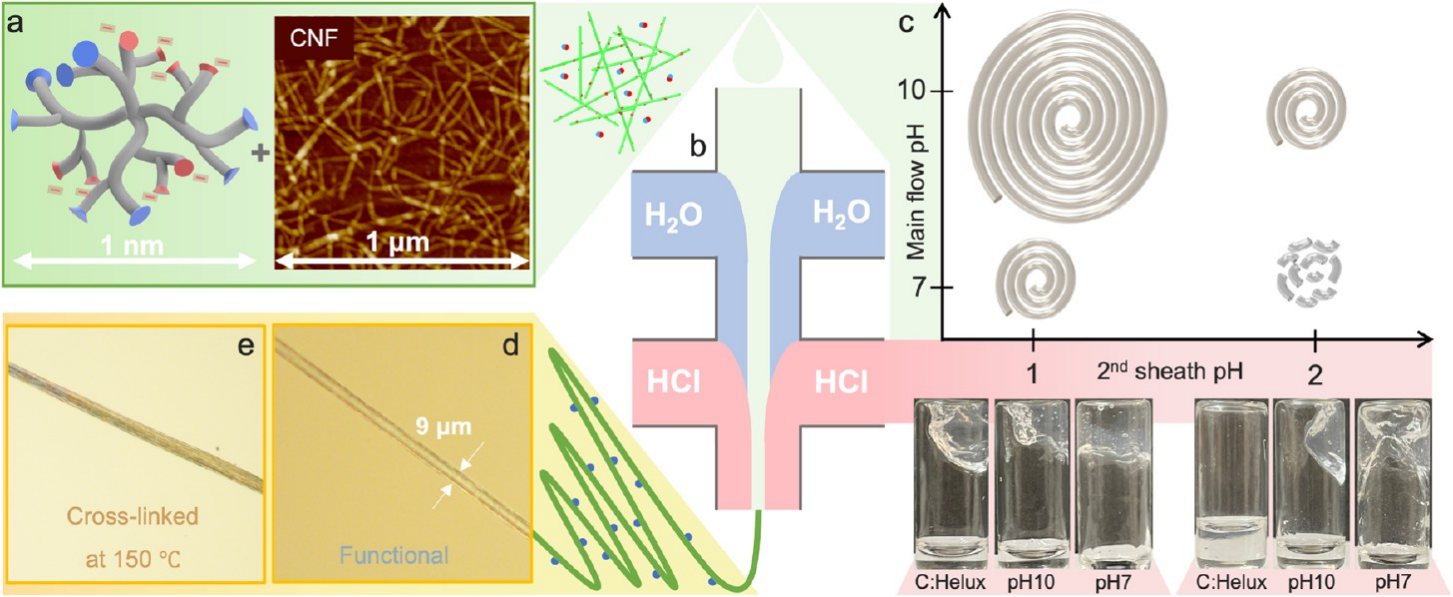
Hydrodynamic assembly of CNF:Helux filaments. (a) Helux (left), consisting of both carboxyl (red sites) and amine (blue sites), mixed with TEMPO-oxidized CNFs (right) flows through the (b) vertical double flow-focusing channel and are aligned by extensional flow (first sheath, blue), forming a gel thread through addition of HCl (second sheath, pink). (c) Spinnability of the composite dispersions at various pH values; Due to the aggregation of Helux and CNFs at neutral pH, the composite dispersion cannot be spun at this condition and form aggregates instead. The pH of a composite dispersion must be within the alkaline range in order for it to be considered spinnable. At the bottom of the figure, inverted-gel tests have been conducted on different samples at two different pH values for the gelling agent. It is apparent that HCl at pH 2 cannot form a solid gel due to the high ionic strength of the composite dispersion (at pH 10). To protonate all carboxyl groups and create a strong gel, the pH of HCl must be adjusted to 1.0. A continuous filament (illustrated by the large spiral) is formed by putting the main flow (composite dispersion) at pH 10 and the second sheath (gelling agent) at pH 1 and the dispersion can be considered spinnable. (d) Functional composite filament with an excess of amine groups. (e) Cross-linked composite filament (heat-treated at 150 °C for 1 h).

The CNF:Helux filaments can also be chemically cross-linked at elevated temperatures (150 °C) and form a self-supporting solid 3D structure (Figure 1d). This structure is readily available for postfunctionalization via amidation chemistry using Helux-accessible amines under aqueous conditions.

The ability of Helux to form ionic interactions with CNFs via carboxyl groups at the C6 position is similar to how hemicellulose binds cellulose fibrils through hydrogen bonding,^6^ forming a flexible and interconnected network. Meanwhile, lignin provides additional cohesion and rigidity, much like Helux, contributing to the mechanical integrity of CNF-based filaments by physically linking fibrils and creating a denser morphology. These similarities suggest that Helux mimics the key structural roles of lignin and hemicellulose, potentially influencing the mechanical properties of the composite filaments.

## Experimental Section

### Preparation of CNF

CNF dispersions were prepared from never-dried sulfite softwood pulp (Domsjö dissolving pulp from Domsjö Fabriker AB, Sweden) provided by the RISE Research Institute of Sweden. To prepare CNFs, never-dried pulps were first chemically treated with 2,2,6,6-tetramethylpiperidinyl-1-oxyl (TEMPO)-mediated oxidation reactions, as described elsewhere.^16^ Then, the oxidized pulps were thoroughly washed with DI water by filtration. The resultant aqueous dispersion was passed through a high-pressure Microfluidizer (M-110EH, Microfluidics Corp.) 5 times (1 pass through 400/200 μm and 4 passes through 200/100 μm wide chambers connected in series) at 1600 bar at 1 gr l^–1^. The gel-like dispersion was further diluted by adding DI water and homogenized by a mechanical mixer at 12,000 rpm for 10 min (Ultra Turrax, IKA, Germany). Then, the diluted dispersion was sonicated for 10 min (Sonics Vibracell) and centrifuged at 5000 rpm for 60 min to remove precipitates. Eventually, a gravimetric method was used to determine the dry content of the dispersion. The length and diameter of TEMPO-oxidized CNFs were measured using atomic force microscopy (AFM, Dimension 3100 SPM, Veeco) were 100–1200 nm and 1–5 nm, respectively (Figure S1c,d). The surface charge density of fibrils was assessed by conductometric titration^17^ and was around 1 μeq g^–1^.

### Preparation of Composite CNF:Helux Dispersions

The 50 wt % aqueous dispersion of heterofunctional hyper-branched poly­(amido amine) carboxylate (Native Helux) (Figure S3) was kindly provided by Polymer Factory Sweden AB. Since Helux comprises primary and secondary amines as well as carboxylic acids, it is pH-dependent and considered as a polyampholyte.^14^ The provided Helux was in its anionic form (pH 10.5) and could easily mix with colloidal dispersions of CNF in a wide range of ratios.^15^ To avoid any coacervation before spinning and have a flowing composite dispersion, the pH of the CNF dispersion was adjusted to 10.5 using NaOH, and Helux was subsequently added to the dispersion and mixed for 15 min using a magnetic stirrer.

### Flow Setup

Three syringe pumps (WPI, Aladdin-2000), one double flow-focusing channel, and a water bath are used to conduct the flow experiment (Figure S4). The CNF suspension is transferred into the core flow of the channel. DI water is transferred to the first sheath to detach the core flow from the walls and assist in the alignment of fibrils. Since the ionic strength and pH of suspension mixtures strongly affect the interaction between ammoniums on Helux and carboxylates on CNFs,^18^ HCl at pH 1 is injected as the second sheath (located 5 mm downstream from the first sheath) flows to trigger gelation and lock the aligned CNFs (at pH 10). Core, first, and second sheath flow rates are 4.1, 4.4, and 24.6 mL h^–1^, respectively. A 1 mm thick stainless steel plate was milled to form the channel, which was sandwiched between two Plexiglas plates. In order to prevent leaks, stainless steel plates were screwed on either side. The channels were each 1 mm wide. The channel outlet was submerged in DI water bath. Hydrogel threads of the composite were picked out with tweezers followed by air drying for at least 2 h at room temperature. Then, the hydrogel threads were dried to form solid composite filaments.

### Cross-Linking of Composite Filaments

CNF:Helux (100:5) filaments were spun using a hydrodynamically induced assembly method. Then, the filaments were glued to U-shaped paper holders. Eventually, they were heated in an oven at 150 °C for 1 h, after which the cross-linked composite filaments could be achieved.

### Tensile Test

The tensile tests were carried out by means of an Instron E1000 instrument equipped with a 0.5 N load cell. Filaments were conditioned at room temperature (23 °C) and 50% relative humidity for at least 1 day before conducting measurements. The diameter of each filament was measured using an optical microscope (Carl Zeiss Axioplan) with a camera (ProgRes Jenoptik, Jena), and a benchtop Dino-Lite digital microscope was used to evaluate its length. For a few samples, cross sections were further cross-checked with SEM; they were uniform throughout the length and assumed to be circular. The diameter may vary a bit between different samples, but it is around 8.5 μm for both the CNF and composite filaments on average. Filaments were first glued on U-shaped paper holders and then clamped between the grips of the tensile test instrument. The gauge length was 12 mm, and measurements were carried out at a cross-head speed of 0.5 mm min^–1^. Tensile test results were averaged over at least 20 different filaments for each sample.

### Cross-Polarized Optical Microscopy Flow-Stop

The experimental setup of POM flow-stop is illustrated in Figure 6a.^19^ In brief, a flow-focusing channel consisting of four crossing channels having a square cross section of side *h* = 1 mm is placed between two 140 mm thick transparent cyclic olefin copolymer (COC) films (Tekni-plex 8007 X-04) due to its low birefringence and beneficial optical properties. Two 10 mm aluminum plates are placed outside the COC films and clamped with screws for the mechanical support. The main flow in the central channel (flow rate *Q*_1_) is focused by two perpendicular distilled water sheath flows (flow rate *Q*_2_/2 each) and accelerated leading to an increased alignment of the nanofibers along the flow direction.^8^ CNFs remain nonisotropic throughout the visible region when only water is run through the second sheath flow, without causing gelation. The *Q*_1_ = 23.4 and *Q*_2_ = 27 mL h^–1^ are selected as flow rates for this experiment.^8^ Using the birefringence properties of nanofibers, their relative orientation in the flow can be measured.^19,20^ The flow cell is placed in front of a high-speed camera (Mako U-029B) to record the intensity of red light generated by the laser module (130 mW and wavelength of ≈660 nm) and transmitted through the system. Images at a rate of 1000 per second are captured for 15 s after the flow is stopped where the first 5 s are enough for flow to reach a stationary state.

### Electron Microscopy Characterization

Surface structure and cross section of filaments were analyzed using a Hitachi S-4800 field emission scanning electron microscope operating at an acceleration voltage of 1 kV. Filaments were sputter-coated with a few nanometer-thin gold*-*palladium layers (Gressington Instruments Ltd., UK) before observations. For cross section analysis, samples were sputter-coated with the same material and method after tensile testing.

### Rheology

Rheology measurements to investigate the viscoelastic properties of CNF and CNF:Helux dispersions were carried out using a 702 e Space rotational rheometer (Anton Paar, Graz, Austria) with a 25 mm diameter parallel plate setup and a gap of 500 μm. The temperature was held constant at 25 °C using a C-ETD200/XL measuring cell. Flow sweep measurements were performed to assess the shear viscosity behavior of the colloidal dispersions in the range 10^–2^ to 10^2^ s^–1^.

### In Situ Microbeam Small-Angle X-ray Scattering (μSAXS)

The μSAXS experiments were performed at P03 beamline at PETRA III storage ring at Deutsches Elektronen-Synchrotron (DESY) in Hamburg, Germany.^21^ The experiments were carried out in a single flow-focusing channel and without gelation due to the long exposure times and limited access to the experimental hutch. In order to allow for comparison, the flow rates were chosen to be the same as in previous work:^8^ *Q*_1_ = 23.4 and *Q*_2_ = 27 mL h^–1^.

The channel was cut from a stainless steel plate of 1 mm thickness and sandwiched between two Kapton films (DuPont, 200HN, each 51 μm thick) instead of Plexiglas (Figure 8a). The complete cell is equipped with dual outer aluminum plates to ensure the mechanical stability and efficient distribution of fluids. Measurements were carried out using a transmission geometry at an X-ray wavelength of λ = 1.05 Å and a sample-to-detector distance of 5.3 m. The beam size was 22 × 13 μm^2^ (horizontal × vertical), and a single-photon counting detector (Pilatus 2 M by Dectris, Switzerland) having a pixel size of 172 × 172 μm^2^ was used to monitor the scattering diffractograms. Similar to previous studies, order parameters were calculated from μSAXS scattering patterns to quantify fibril alignment.^8,22^ μSAXS patterns were quantified by first transforming the diffractogram into a rectangular image containing the scattering vector *q* (defined as *q* = 4π sin­(θ)/λ, where θ is the angle between incident and scattered light) and azimuthal angle, *χ*, as coordinates. For each *q*-value, the background intensities (only DI water flowing) were subtracted assuming that fibrils aligned perpendicular to the flow (the value at *χ* = 90 was set to zero) did not exist in the highest oriented case. In each azimuthal angle, the final intensity (orientation) distribution was averaged between 0.2 < *q* < 0.8 nm^–1^. The quantification of CNF alignment was achieved by converting the orientation distributions into order parameters (*S*):

1 $$S_{\chi }=\left\langle \frac{3}{2}\;\cos^{2}\chi -\frac{1}{2}\right\rangle$$

where *χ* is the azimuthal angle in a diffractogram (on a plane perpendicular to the X-ray beam). Expanding the average gives

2 $$S_{\chi }=\int_{0}^{\pi }I\left(\chi \right)\left(\frac{3}{2}\;\cos^{2}\chi -\frac{1}{2}\right)\;\sin\chi d\chi$$

which is normalized according to

3 $$\int_{0}^{\pi }I\left(\chi \right)\;s\mathrm{in}\;\chi d\chi =1$$

where *I*(*χ*) is the intensity distribution along a constant *q*-value for each azimuthal angle.

### Wide-Angle X-ray Scattering (WAXS)

The WAXS measurements were also carried out at PETRA III storage ring (P03 beamline) at DESY in Hamburg, Germany.^21^ The shift in focus from the analysis of alignment of cellulose nanofibers in the flow at a larger scale to the examination of alignment of crystal planes in the dried filaments at a smaller scale can be achieved by moving the detector closer (sample-to-detector distance of 205 mm) to the sample (compared to SAXS) and investigating wider angles representing smaller distances inside the sample *l* = 2π/*q* = λ/2 sin (ζ/2), where ζ is the scattering angle. To monitor the scattering diffractograms, an X-ray with a wavelength of λ = 1.05 and a beam size of 27 × 25 μm^2^ (horizontal × vertical) counting LAMBDA 9 M detector system (X-Spectrum GmbH, Germany) with a pixel size of 55 × 55 μm^2^ was used. For each sample, a bundle of ten filaments with a diameter of ≈40 μm was created and placed on the beam trajectory. For each *q*-value, the background intensities (from air) were subtracted. In each azimuthal angle, the final intensity distribution was averaged within the *q*-range of 13 < *q* < 17 nm^–1^. Taking into consideration the intensity distribution profiles and azimuthal integration over 200 crystal lattice *q* ranges, since the cellulose crystals are aligned in the fibril direction,^8^ it is possible to derive the orientation index *f*_c_ from the azimuthal scattering variation as follows:^23^

4 $$f_{c}=\frac{180^{{}^{\circ}}-\mathrm{FWHM}}{180^{{}^{\circ}}}$$

where fwhm is the full width at half-maximum of the azimuthal integration over the 200 crystal lattice scattering peak.

### Ninhydrin Assay

The primary quantification of amines in CNF:Helux filaments was performed by using the ninhydrin test. The ninhydrin test protocol was modified from a previously published procedure.^24^ To a solution of ninhydrin (300 mg) and hydrindantin (45 mg) in anhydrous dimethyl sulfoxide (DMSO) (11.25 mL) was added lithium acetate buffer, pH 5.3 (3.75 mL). The solution was then deoxygenated. CNF:Helux filaments (60 mm, Figure S6) suspended in Milli-Q water (1 mL) and ninhydrin solution (1 mL) were placed in a sealed vial and heated to 100 °C for 15 min. 50% water/EtOH solution (3 mL) were added to the reaction mixture, filtered through a 0.45 μ pore filter and absorbance at λ = 570 nm measured. N-Butylamine solutions in a concentration range of 10–200 μM were used as the calibration. The calibration curve of N-butylamine solutions at absorbance at λ = 570 nm is provided in Figure S7.

### Chemical Postfunctionalization Of Composite Filaments

Disperse red succinic *N*-hydroxysuccinimide (DredS-NHS) (Figure S8) was prepared according to the previously published protocol.^15^ CNF:Helux filaments (60 mm) were suspended in phosphate buffer (pH 7) (1 mL) in an Eppendorf tube. DredS-NHS (500 μL) dissolved in DMSO (1 mg mL^–1^) was added to the composite filament buffer and allowed to react for 10 min before the solid material was washed with an excess of DMSO and H_2_O until considered free of physically adsorbed dispersed red. Optical microscopy was used to visualize the dye effect where dyed CNF:Helux filament was compared to dyed CNF filament.

## Results and Discussion

### Stability, Flowability, and Spinnability of CNF:Helux Composite Dispersions

In a double flow-focusing spinning channel (Figures 1b and 2), a core flow is focused by sidestreams (sheath flows). The polydisperse CNFs in the core are aligned by extensional flow and then undergo an ion-diffusion-based sol–gel transition induced by ions from the sheath flows forming a thread.^25,26^ The mechanical performance of filaments produced by this process relies heavily on the alignment of the fibrils and interfibrillar interactions. The most critical parameter controlling this is the fibril concentration.

**Figure 2:**
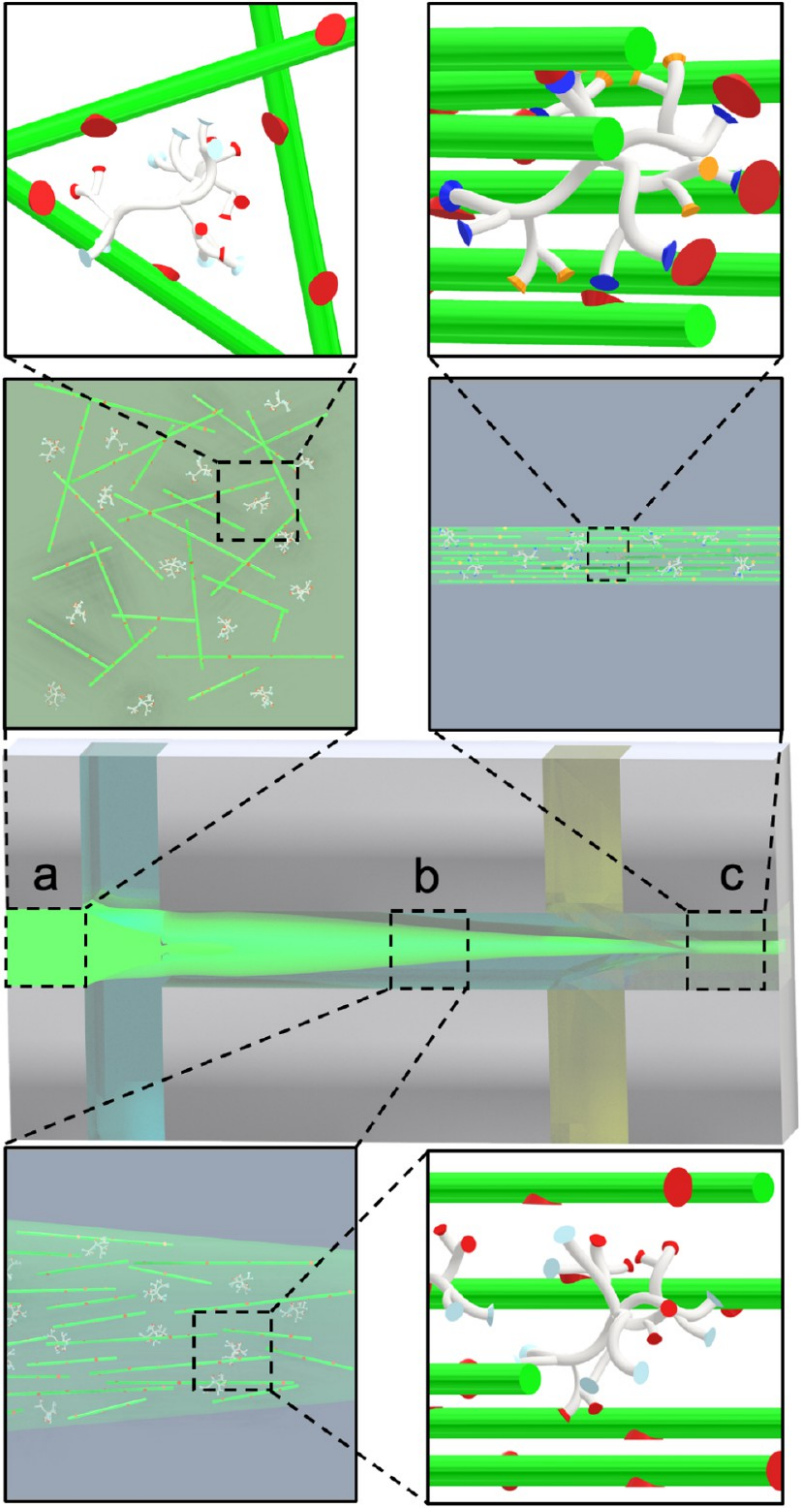
Illustration of the alignment and gelling. (a) At pH 10, all Helux amine groups (light blue sites) are neutral, making it possible to mix it with CNFs without coacervation and inject their composite dispersion into the main inlet channel (horizontal channel). (b) CNFs and Helux particles repel each other while aligning toward the flow direction due to extensional flow. (c) All Helux amine groups are protonated (ammonium groups, dark blue sites) at pH 1 and connected to several CNFs through carboxyl group neutralization (physical cross-linking).

On one hand, the relatively high concentration and broad size distribution of CNFs lead to interfibrillar interactions that restrict their nanoscale alignment within channel. A large number of CNFs in a confined area results in a high crowding factor $N=\frac{2}{3}\phi {\left(A\right)}^{2}$ , where ϕ is concentration and *A* is aspect ratio. In a highly crowded environment (*N* > 60), CNF alignment is limited and their motion is hindered by neighboring CNFs. The ideal concentration for free rotation is slightly below the liquid-glass transition concentration (*C*_glass_, rigidity threshold *N* = 60).^27^ At this concentration, free volume and particle mobility are constrained, as each fibril is locked by about 3 contact points (*n*_c_ = 3), leading to a solid-like reversible gel with repulsive interactions.^28^

On the other hand, to form an interconnected percolated 3D fibril network (gel thread), the concentration must be slightly above the connectivity threshold (*C*_conn_, *N* = 16),^29^ corresponding to a solid-like (*G*′ > *G″*) volume spanning arrested state (VAS). Above *C*_conn_, a nonconnected percolated network (*n*_c_ = 2) forms, which restricts fibril translation but still allows some rotation and diffusion along their axis.^30^ Thus, colloidal stability is necessary but insufficient for the hydrodynamic assembly of CNFs. For a dispersion to be spinnable, its concentration *C*_spin_ must satisfy *C*_conn_ < *C*_spin_ < *C*_glass_ and depends on the fibrils’ aspect ratio and length distribution.

When it comes to a composite dispersion, this concentration range would shift to lower or higher ranges depending on the structure of the second particle and its effect on the viscosity and flow behavior of the CNF dispersion. The dendritic polyampholyte—Helux—used in this study is a small molecule with a condensed, hyper-branched structure (radius of gyration *r*_g_ = 1 nm) (Figure 1a), with both anionic and cationic sites (red and blue sites in Figure 1a). It can be mixed with CNFs at a concentration in the vicinity of *C*_conn_ without any coacervation (at pH 10).^15^ Despite the anionic nature of Helux, at *C* > *C*_conn_, it is absorbed on CNFs and decreases the repulsion between them, resulting in thixotropic behavior. Thus, *C*_glass_ is lower for the CNF:Helux dispersion compared to pure CNF and, as a consequence, the spinnability window is narrower. As found previously,^15^ due to the deprotonation of the carboxylic acids at elevated pH (in our case 10.5), Helux can be mixed with 0.2 wt % CNF (0.6 μeq g^–1^) with the weight ratio of 5:100 forming a stable flowing composite dispersion. Based on inverted-gel tests, it is found that the dispersion is no longer flowable neither at higher CNF:Helux ratios (≥10:100) nor at higher CNF concentrations (≥0.3 wt %). Therefore, we have mixed 0.2 wt % (1 μeq g^–1^) CNF dispersion and Helux with a 100:5 ratio to ensure that the dispersion is in the spinnability window and the fibrils have enough freedom to move (rotate, translate, and diffuse) and align in the flow. The atomic force microscopy (AFM) imaging of CNFs adsorbing onto a silicon wafer from a mixture of 100:5 CNF and Helux (net anionic) also shows the fibrils in a nonaggregated state since individual CNFs with widths of ∼3 nm were observed (Figure S1a,b). Summarizing, we will compare three dispersions as detailed in Table 1.

**Table 1:** Summary of Dispersions Used for Filament Spinning and Their Spinnability

	CNF concentration	dispersion	CNF:Helux	2nd sheath		
dispersion	wt %	pH	ratio	pH	spinnability	name in manuscript
CNF (1 μeq g^–1^)	0.2	7.0		1.0	continuous thread	CNF pH 7
CNF (1 μeq g^–1^)	0.2	10.5		1.0	continuous thread	CNF pH 10
CNF:Helux	0.2	7.0	95:5	2.0	agglomerates	
CNF:Helux	0.2	7.0	95:5	1.0	short thread	
CNF:Helux	0.2	10.5	95:5	2.0	short thread	
CNF:Helux	0.2	10.5	95:5	1.0	continuous thread	CNF:Helux

From a rheological perspective, all of the dispersions are shear thinning (see Figure S5), as is typical for CNF dispersions. At a fairly low shear rate γ̇ ≈ 0.03 s^–1^, the viscosity (η) of the CNF:Helux dispersion decreases faster than the reference (pH 7), and at γ̇ ≈ 0.2 s^–1^ the same observation is made for the pH 10 case. At γ̇ > 1 s^–1^, the CNF:Helux and pH 10 cases have a viscosity that is approximately 50% and 80% of that of the pH 7 case, respectively. This indicates that the rotary dynamics (quantified by the rotary diffusion *D*_r_ discussed later) of the fibrils is faster in the pH 10 and CNF:Helux cases, as compared to the pH 7 reference.

### Mechanical Properties and Alignment in Filaments

The mechanical performance of filaments is strongly correlated to nanostructure alignment and the interaction between fibrils. As explained by e.g., Doi et al.,^31^ in a dispersion of nonspherical particles, alignment (and consequent mechanical performance of assembled filaments) is an integrated result of: (i) velocity gradients causing particle rotation, (ii) Brownian diffusion, and (iii) particle interactions through direct contact, electrostatics and/or hydrodynamics. CNFs are semiflexible fibers that have a higher degree of flexibility and ability to bend than other nonspherical particles, such as rigid rods or platelets. Consequently, there are greater orthogonal fluctuations in their network structures. In contrast to rigid nonspherical particles, which form static, predictable structures, CNFs create dynamic networks with adjustable pathways and varying confinements. Due to their semiflexible nature, CNFs exhibit distinct mechanical properties and network behaviors compared to rigid nonspherical particles.^32^

To elucidate how the pH, addition of Helux, and cross-linking affect the mechanical performance of filaments, we prepared CNF (at pH 7 and pH 10) and CNF:Helux filaments for the tensile test. Chemical cross-linking of composite filaments was achieved by heat treatment at 150 °C for 1 h. A typical stress–strain curve per sample is shown in Figure 3a. All of the filaments exhibited both pseudoelastic (linear region) and plastic (deviation from linearity) behavior. Pure CNF filaments showed almost the same initial stiffness at different pH values. There is a transition from elastic to plastic deformation at a yield point of around 1% strain. This is attributed to the unrecoverable interfibrillar debonding/slippage.^33^

**Figure 3:**
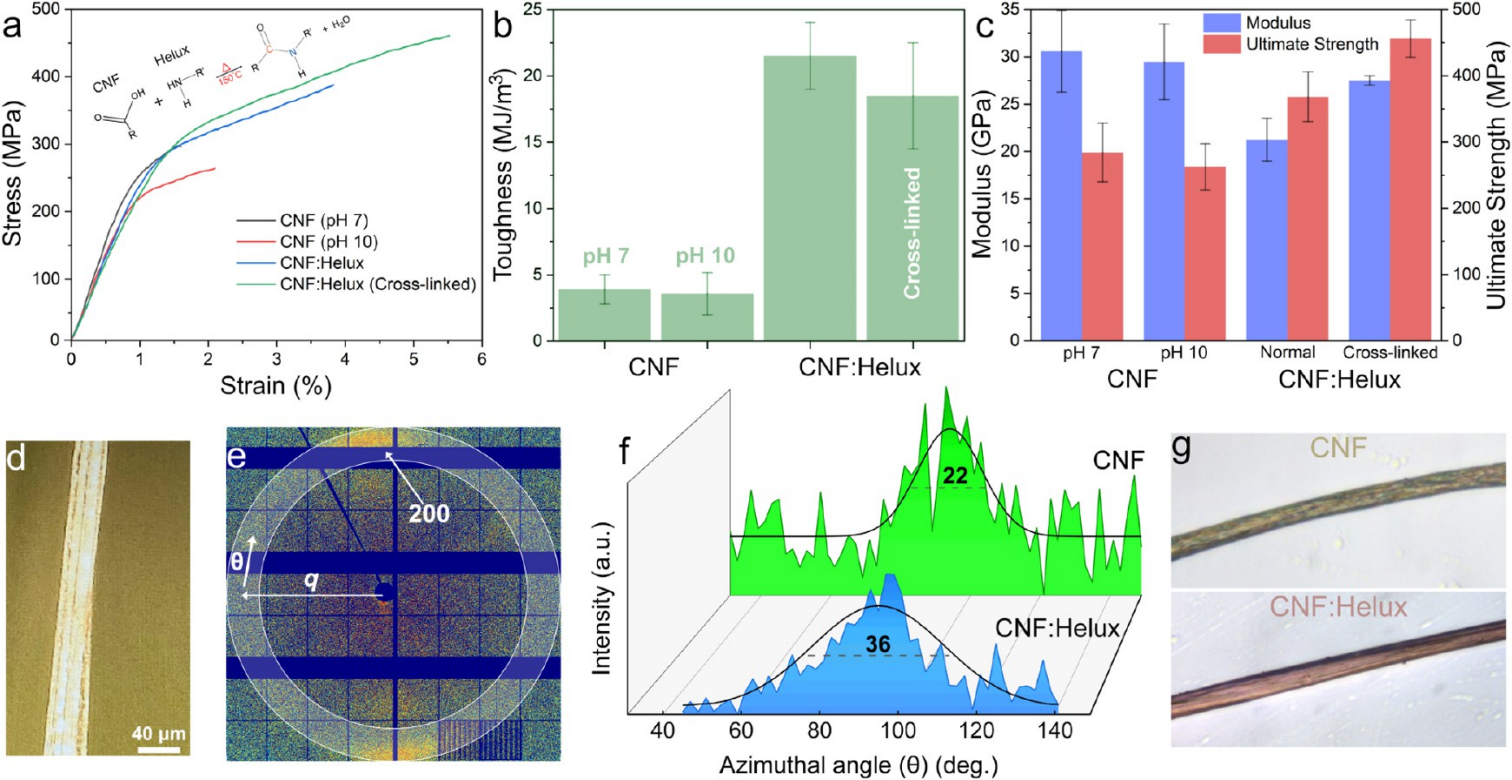
(a) Representative stress–strain curves of filaments made from CNFs and Helux indicating the influence of pH, Helux, and its cross-linking on the mechanical performance of filaments prepared from a double flow-focusing channel measured at 50% relative humidity (RH). The cross-linking of CNFs and Helux occurs via the formation of amide bonds between the ammonium groups of Helux and the carboxylate groups of CNFs at elevated temperatures.^15^ (b) Toughness of filaments. (c) Modulus and ultimate strength of pure CNF (at pH 7 and pH 10) and composite filaments. (d) A bundle of ten filaments with a diameter of ∼40 μm was created and placed on the beam trajectory. (e) Diffractogram corresponding to the composite filament. (f) Azimuthal integration of the diffractograms for CNF (green) and CNF:Helux (blue) filaments in the (200) scattering plane. The black lines represent Gaussian fits to the data, while the dashed lines indicate the full width at half-maximum (fwhm) values, which are also labeled in the plot. (g) Dred-NHS-modified CNF and CNF:Helux filaments; CNF:Helux filament displaying the characteristic color of the dye as a result of interaction with accessible amines of the Helux.

The plastic region is more pronounced for composite filaments, indicating that CNF and Helux form stronger 3D networks than pure CNF. This can be attributed to physical bonding between the carboxyl groups of CNF and the amine groups of Helux.^15^ The reduced Debye length of CNFs in the presence of Helux^34^ further supports this, as Helux screens the carboxylate groups more effectively than H^+^ ions from hydrochloric acid (HCl) due to its larger size and higher valence. Higher valence and counterion concentration enhance double-layer screening,^35^ following the Schulze-Hardy rule, where the screening threshold is proportional to *z* ^6^, where *z* is the valence of the counterion.^36^ Cations with higher valencies can also form more rigid hydrogels, shaping a stronger 3D network structure in rheology measurements.^37^

As previously reported,^38^ the strength of CNF hydrogel correlates with the electronic structure of the gelling agent and its binding strength to the carboxyl groups. From an entropic perspective, multivalent ions promote faster gelation by providing electrostatic shielding, similar to that of several monovalent ions. The high valency of Helux enhances fibril association, fostering the formation of a physical 3D network through cross-linking (physical). Though rare due to steric hindrance, Helux can bridge fibrils, cross-linking adjacent chains on the same fibril and interacting with carboxyl groups on others, given the distance between surface molecular chains (0.53–0.61 nm) on a fibril.^39^

The area under the stress–strain curve is used to calculate the toughness of the material, which describes how much energy the material absorbs per unit volume during deformation before failure. The achieved toughness was roughly six times higher for composites compared with pure CNF (Figure 3b). However, the degree of alignment is lower in composite filaments. Nevertheless, due to stronger 3D structures between CNFs and Helux, the plastic region is much larger compared to the CNF alone (Figure 3a). Thus, high orientation does not necessarily equate to high toughness.^40^

Moreover, composite filaments exhibited higher ultimate strength (Figure 3c) compared with CNF filaments. Cross-linked composite filaments showed the highest ultimate strength since the heterofunctionality of Helux enables the formation of irreversible covalently cross-linked 3D networks.^15^ A covalent amide bond is formed between CNF carboxyl groups (red sites in Figure 1) and Helux amine groups (blue sites in Figure 1) during cross-linking at an elevated temperature. An increase in the modulus and ultimate strength has also been reported previously after chemical cross-linking.^41^ This interaction mechanism is comparable to natural wood, where cellulose, a linear polysaccharide, is cross-linked by hemicellulose (a two-dimensional polymer) and reinforced by lignin (a three-dimensional macromolecule).^6^ Just as hemicellulose and lignin enhance mechanical properties of the wood, Helux improves filament toughness and strength by forming multivalent interactions with CNFs, reducing voids, and reinforcing the network.

As can be observed in Figure 3c, the lower elastic modulus (21 GPa) in composite filaments could be attributed to the decrease in fibril orientation in the presence of Helux observed by wide-angle X-ray scattering (WAXS) (Figure 3e). The value of the orientation index for CNF:Helux filaments (*f*_c_ = 0.8) is slightly lower than the value for CNF filaments (*f*_c_ = 0.87). These values explain the lower modules of uncured CNF:Helux compared to the pure CNF filaments.

The surface morphologies of both CNF and composite filaments are shown in Figure 4a,c. In comparison with pure CNF filaments, composite ones have a more tightly packed structure, which is in agreement with the tight structure of CNF hydrogels formed in the presence of high-valency ions.^42^ As a multivalent cation (under acidic conditions), Helux exhibits stronger intra- and interfibrillar interactions than H^+^ cations. Ammonium groups on Helux (in the gelled state at low pH) hinder carboxylate groups on cellulose from electrostatically repelling each other, resulting in a denser structure. However, fibrils form less aligned structures in the presence of Helux due to (i) higher mobility of fibrils and faster dynamics at elevated pH and (ii) amplified electrostatic repulsion from carboxylate groups on Helux.

**Figure 4:**
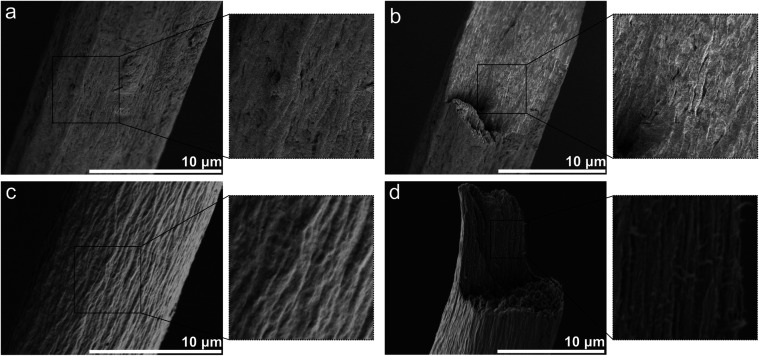
(a) Surface morphology and (b) fracture surfaces of CNF filament. (c) Surface morphology and (d) fracture surfaces of CNF:Helux filament.

Pure CNF filaments have voids and ridges caused by the loose packing of fibrils where carboxyl groups are protonated with small hydrogen ions and become electrostatically neutral, illustrated by the blue spheres in Figure 5. Similar surface features have been observed on other filaments made from CNF recently.^8,22,25^ In contrast, a Helux molecule in a composite filament can ionically interact with multiple fibrils and physically link them together; at elevated temperatures (150 °C), these interactions can be further strengthened through the formation of amide bonds as schematically indicated in Figure 5. By creating a 3D structure, the surface of the filament becomes smoother and its performance under mechanical loads improves.^9^

**Figure 5:**
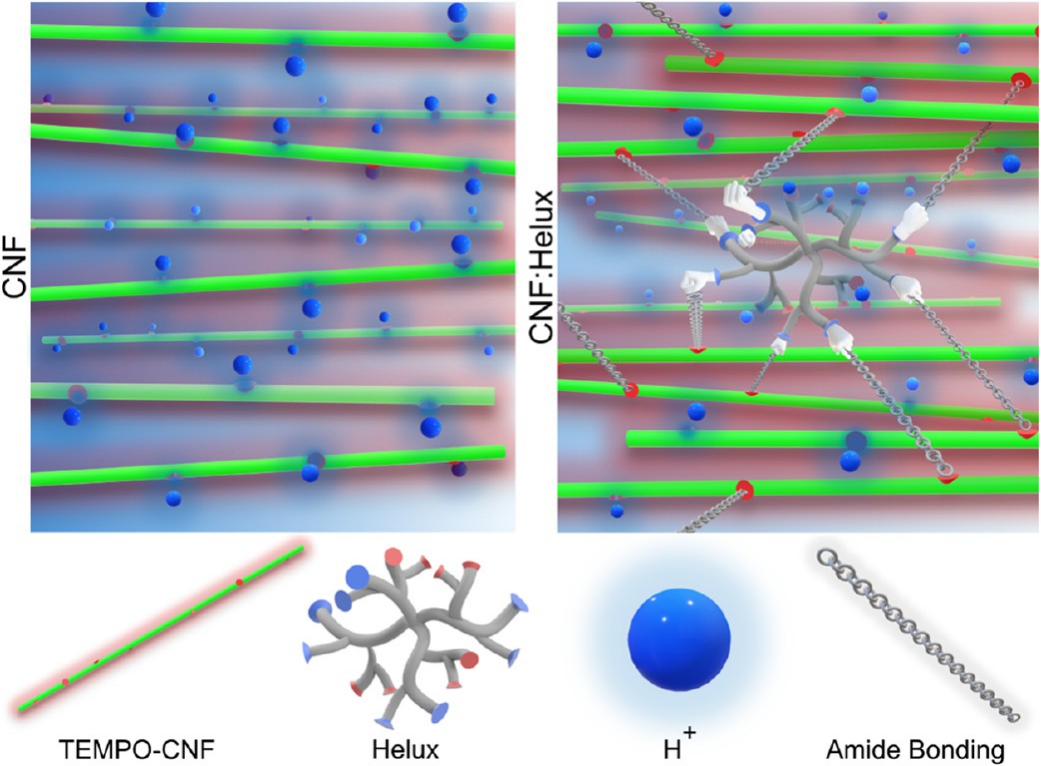
Schematic representation of pure CNF filaments (left) and CNF:Helux composite filaments (right). In pure CNF filaments, loose fibril packing leads to surface voids and ridges. Helux enhances interfibrillar interactions, promoting densification and reducing alignment. At elevated temperatures (150 °C), Helux facilitates amide bond formation, further strengthening the filament structure.

Figure 4b,d depicts the morphologies of the tensile-fractured surfaces of CNF and CNF:Helux filaments. The voids and loosely arranged fibrils in CNF filaments may result in a lower level of ultimate strength and toughness.^43^ As a result, poorly connected regions will cause structural defects in the final structure and may lead to premature failure of the material.^40,41^ Cellulose nanomaterial toughening is an extrinsic phenomenon because of the pull-out process restrained by fibril bonding.^43^ As observed, the tensile-fractured surfaces of CNF exhibit cleavage planes and flat regions with minimal plastic deformation, showing no evidence of fiber pull-out or elongation through the fibers (Figure 4c). Fractures occur more abruptly as a result of this. In contrast, the CNF:Helux filament (Figure 4d) exhibits very dense packing and a rougher tensile-fractured surface, which deformed ductilely. Surfaces of tensile-fractured composite filaments show signs of fibrillation or elongation, where fibers may have pulled out of the matrix, leaving voids. This is the mechanism behind the large plastic region in Figure 3a, which absorbs a lot of energy before the final failure.

The addition of Helux introduces a delicate balance between the alignment and mechanical performance. While fibril alignment is typically a key driver of high stiffness and strength, Helux-mediated interactions promote enhanced interfibrillar bonding and densification, which compensate for the loss of alignment by fostering a tougher, more resilient network. This suggests that mechanical optimization is not solely dependent on alignment but also on fibril interactions, similar to the balance observed in natural wood, where hemicellulose-lignin interactions enhance structural integrity.^44^ These interactions are crucial for understanding and engineering the strength, toughness, and functionality of cellulose-based materials. Nevertheless, the modulus of filaments depends prominently on the orientation of fibrils rather than interfibrillar interactions holding them together under the tensile load.

### Fibril Alignment I: Birefringence and Flow-Stop Measurements

Due to the coupling between mechanical properties and fibril alignment observed in the previous section, fabrication of strong functional macroscale structures requires an in-depth understanding of nanoscale fibril dynamics. Alignment of fibrils not only improves elastic modulus but also facilitates interfibrillar interactions by bringing them closer to each other.

During assembly, there is a competition between thermal fluctuations causing translational and rotational diffusion (Brownian motion) and alignment induced by hydrodynamic forces inside the microfluidic channels.^20^ On the one hand, a comprehensive analysis of rotational diffusion in a polydisperse system of CNFs is crucial for optimization of macroscale properties either by (i) controlling parameters such as concentration, viscosity, etc., or by (ii) introducing a polyelectrolyte complex contributing to the formation of three-dimensional network structures. On the other hand, having a polydisperse length distribution of nanofibers has a significant effect on the nanoscale entanglement that restrains Brownian relaxation toward isotropy upon reduction of aligning velocity gradients during hydrodynamic assembly.^45^

The analysis of orientation dynamics of nanofibers under dynamic flow conditions cannot be carried out using conventional characterization techniques, such as rheology and electron microscopy, which are limited to static observations. Thus, it is imperative to employ an appropriate dynamic characterization method, which relates Brownian diffusion to the fibril’s length distribution so as to provide a comprehensive understanding of physical interlocking during nanostructure fabrication.

The flow-stop methodology involves stopping the system rapidly once it reaches a steady state, and measures the relaxation of particle orientation toward isotropy at various places along the channel depending on the diffusion of system constituents.^19^ Cross-polarized optical microscopy (POM) can potentially be used to assess average angular dynamics in situ. The birefringence of the nanofibers *B* is directly proportional to the square root of intensity *I*_POM_, as well as relative orientation of nanofibers inside the channel *S*_ϕ_, i.e.,

5 $$B\;\propto \sqrt{I_{POM}}\;\propto \;S_{\phi }$$

where

6 $$S_{\phi }=\left\langle \frac{3}{2}\;\cos^{2}\phi -\frac{1}{2}\right\rangle$$

is the order parameter describing flow-alignment of fibrils and ϕ is the angle between the fibril major axis and flow direction.

An example of intensity of transmitted light at steady state flow is shown in Figure 6b for CNF at pH 7 and 10, as well as for composite dispersions. Since the intensity of transmitted light is proportional to the square of the order parameter, it can be inferred that the order parameter decreases as pH increases (the intensity is lower for cases CNF:Helux and CNF (pH 10) as compared to case CNF (pH 7) in Figure 6b). It can be explained by the presence of Na^+^ counterions in the dispersion associated with the addition of sodium hydroxide to raise pH and maintain the anionic state of the Helux. The “screening effect” of counterions may cause fibrils to come closer to each other while still repelling adjacent fibrils due to the presence of carboxylate groups. Furthermore, after the addition of Helux, intensities decreased even more; i.e., alignments decreased. As Helux molecules have a heterofunctional property and are completely anionic at pH 10, they repel nanofibers and do not coacervate with them. These mechanisms of reduced fibril/fibril interaction are seen to lead to reduced viscosity (Figure S5) and reduced alignment (Figure 6b).

**Figure 6:**
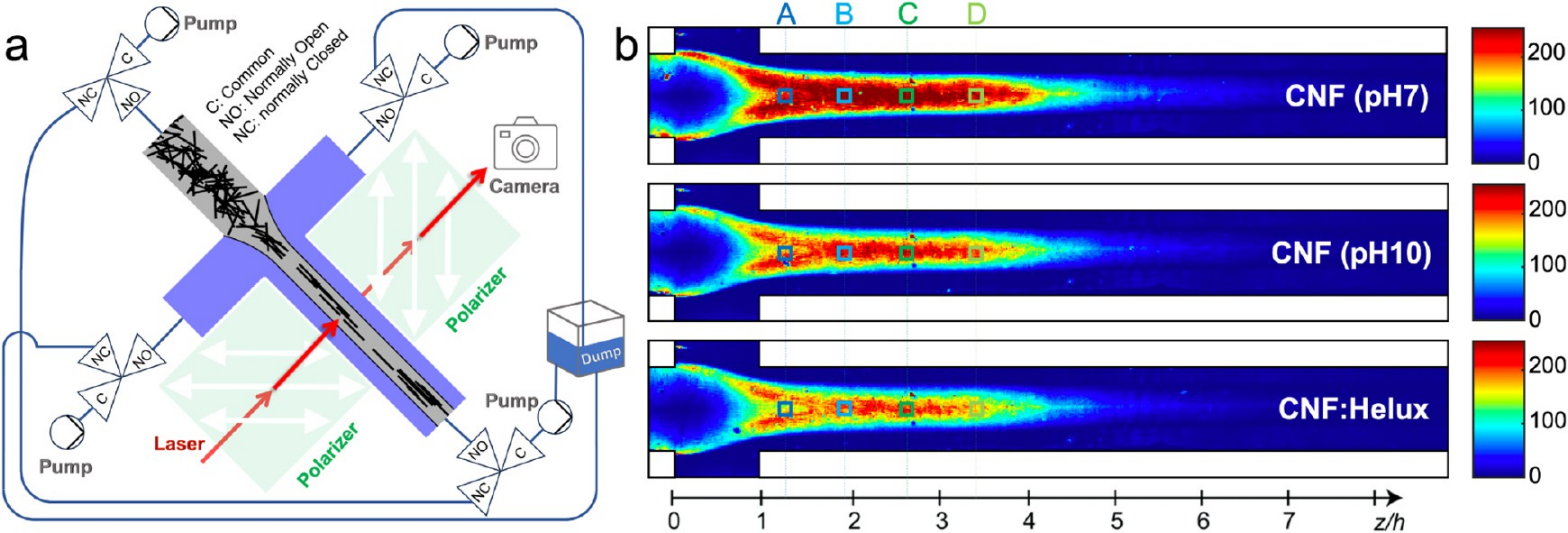
(a) Schematic illustration of the POM flow-stop experiment. (b) POM intensity *I*_POM,0_ during stationary flow in the flow-focusing geometry for CNF (pH 7), CNF (pH 10), and CNF:Helux.

For a more detailed analysis of nanofibers length distribution and fibrils dynamics, the flow was stopped rapidly after reaching a steady state, and the decay of their birefringence was used to assess how they relaxed toward isotropy by Brownian motions. For a monodisperse system, exponential decay of alignment caused by Brownian rotary diffusion corresponds to *S*_ϕ_ ≈ exp (−6 *D*_r_ *t*), where *D*_r_ is the rotary diffusion coefficient. Assuming a dilute system of monodisperse slender nanorods of length *L* in a solvent with dynamic viscosity μ at temperature *T*, *D_r_* is obtained as^31^

7 $$D_{r}\approx \frac{5\;k_{B}T}{\mu L^{3}}$$

where *k*_B_ is the Boltzmann constant.

The highly polydispersed nature of CNF dispersion used in this study implies that relaxation toward isotropy will occur at a wide range of time scales.^20,45^ Shorter time scales are associated with short and less entangled nanofibers, whereas longer time scales are associated with long, partially entangled nanofibers. Thus, postprocessing has been conducted according to the previously reported procedures by Rosén et al.^19^ The exact positions for measurements at different downstream positions are marked in Figure 6b (A to D sections).

Upon closer inspection, two peaks, representing both short and long nanofibers, can be observed at upstream positions (Figures 7a and S2A,B) for all of the samples. At this position, the flow aligns all nanofibers, even those with shortest lengths and fastest rotary diffusion. Thus, the rate of extension *ε̇* is high enough to overcome both the highest and lowest rotary diffusion (*D*_r_max__ and *D*_r_min__) corresponding to short and long nanofibers, respectively (*ε̇* > *D*_r_max__, *D*_r_min__). Further downstream (Figures 7b and S2C,D), long nanofibers with the lowest rotary diffusion *D*_r_min__ are still aligned in all three samples, while short ones with higher rotary diffusion *D*_r_max__ have already been dealigned in both neat CNF dispersions (*D*_r_min__ < *ε̇* < *D*_r_max__), but they are still there for CNF:Helux, indicating *ε̇* > *D*_r_max__. Therefore, transitions of rotational mobility can be detected by a dimensionless Péclet number Pe = *D*_r_/*ε̇*.^45^

**Figure 7:**
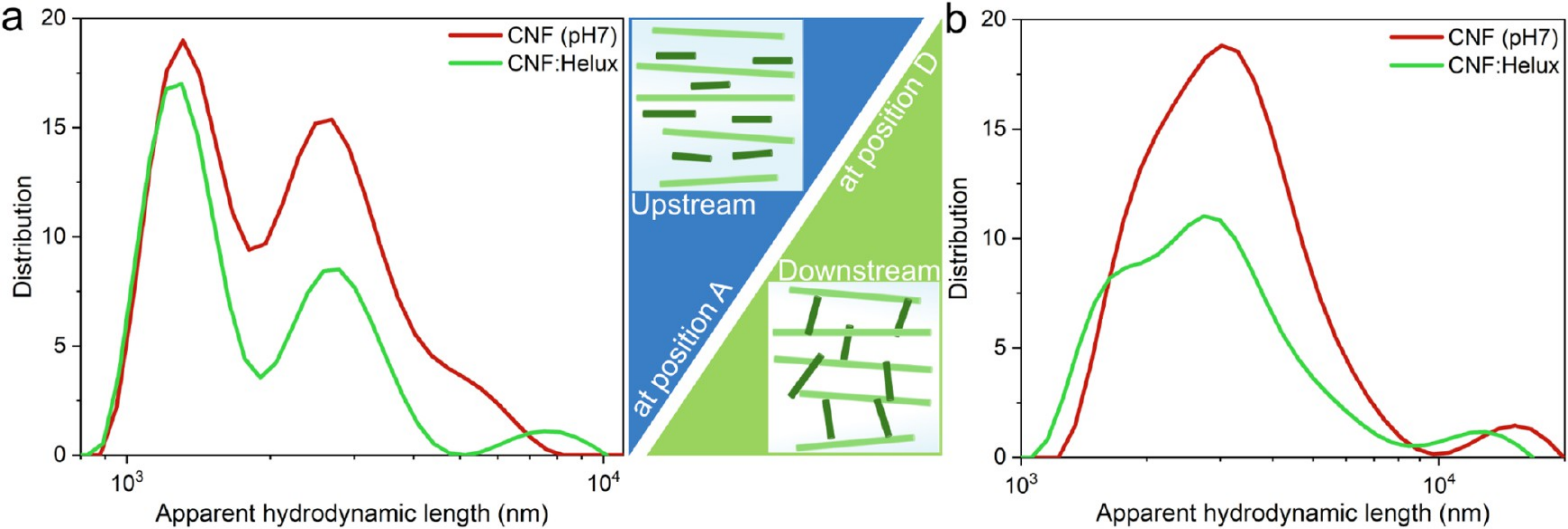
Apparent hydrodynamic length distribution at (a) upstream and (b) downstream positions (based on Figure 6) of flow-focusing channel for CNF (pH 7) and CNF:Helux.

At the downstream position shown in Figure 7b, the curves are shifted to the left (toward shorter apparent hydrodynamic length, i.e., faster rotary diffusion) for CNF:Helux compared to neat CNF. This suggests that the fibrils, which should be similar in both cases, relax *faster* (have higher *D*_r_) in the CNF:Helux case. This can be attributed to repulsive forces between Helux molecules carrying carboxylate groups and CNFs since the electric double-layer interactions strongly affect rotational relaxation.^46^

### Fibril Alignment II: In Situ Microbeam Small-Angle X-ray Scattering (μSAXS) and Fibril Dynamics

The fibril alignment in assembly-like flows is studied (i) experimentally with SAXS in a single flow-focusing channel, where the key elements of the assembly flow (shear at the wall and extensional flow at the focusing) are at hand, and (ii) numerically via simulations of the alignment in the actual double flow-focusing geometry used for filament preparation. The microbeam small-angle X-ray scattering (μSAXS) can be used to monitor fibril alignment in situ by placing the flow setup (Figure 8a) in the beam and obtaining scattering images as shown in Figure 8b. While passing through the channel, X-ray photons scatter anisotropically due to the alignment of CNFs toward flow direction. By normalizing the intensity variation (between 0 and 1) at a given radius (*q*-value) of the scattering images, one can obtain the projected orientation distributions of nanofibers. Then, by traversing the flow channel, measurements at different streamwise positions (Figure 8c) are obtained, and the local order parameter can be calculated (Figure 8d). A value of the order parameter *S_χ_* = 0 refers to an isotropic distribution of fibril orientations (random, before sheath flow in Figure 6a and blue regions in Figure 6b), while *S_χ_* = 1 represents a fully aligned distribution of fibrils (after sheath flow in Figure 6a and red regions in Figure 6b) toward the flow direction.

**Figure 8:**
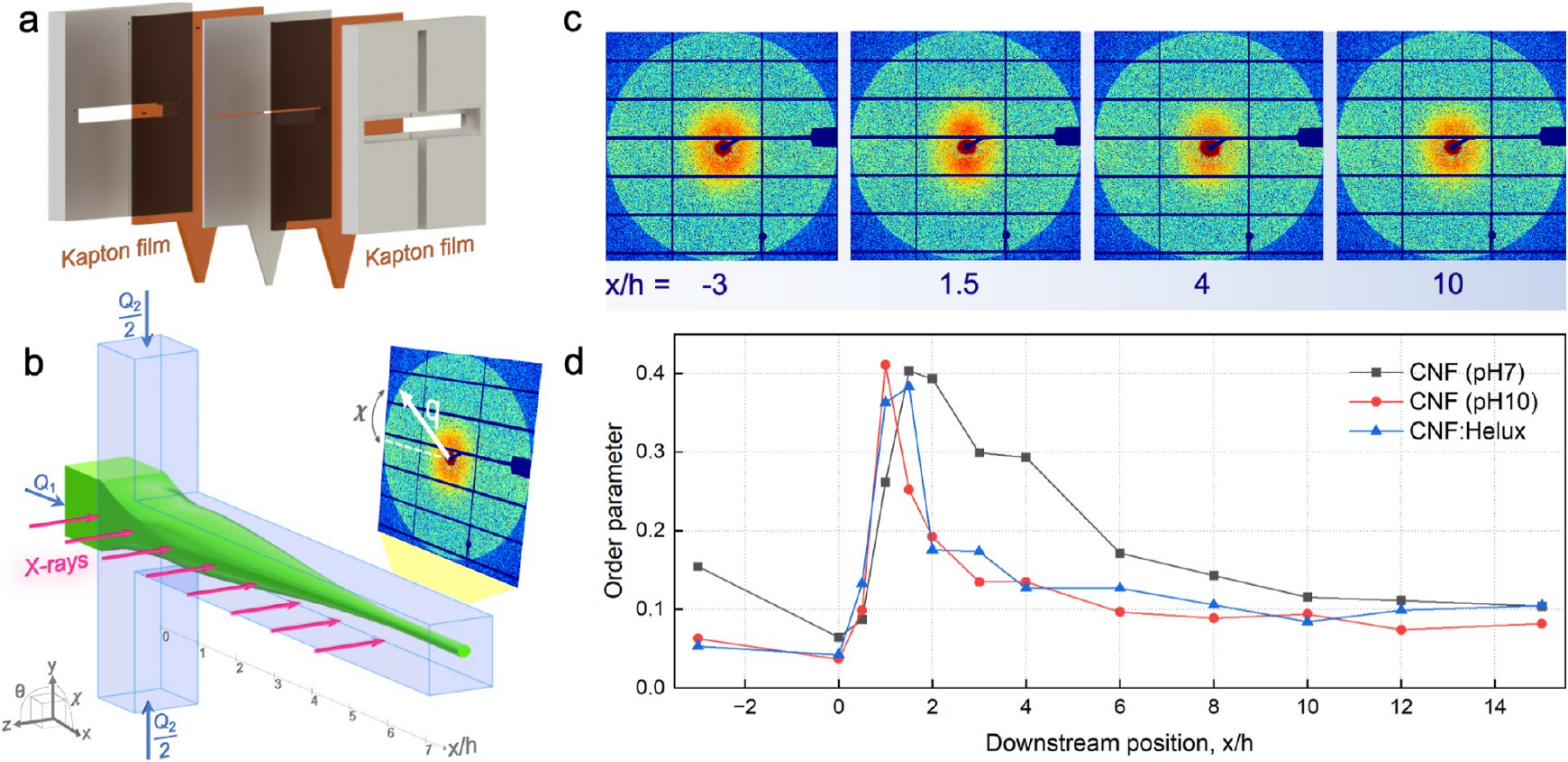
(a) Sandwich structure of the single flow-focusing channel used in the μSAXS experiment. (b) μSAXS setup used to quantify the alignment. (c) μSAXS scattering diffractograms at different positions along the channel for CNF dispersion. (d) Local order parameters calculated from the μSAXS patterns as a function of downstream position *x* in the channel normalized with the channel width *h* = 1 mm.

As seen in Figure 8d, at the beginning (*x*/*h* = −3), the order parameter increases due to the shear forces from the wall, with this effect being more prominent for CNF at neutral pH. Due to deceleration of the core flow, the order parameter decreases at the start of the focusing step (*x*/*h* = 0), followed by an increase after focusing (0 < *x*/*h* ≤ 2). Going further into the details, we can observe that the maximum order parameter for all three samples is roughly the same.

Subsequently, Figure 8d shows that due to Brownian diffusion, the order parameters decreased again for *x*/*h* > 2, indicating that the aligned fibrils are relaxing toward isotropy. The drastic decay in the case of CNF (pH 10) and CNF:Helux is a consequence of the faster dynamics of fibrils in these dispersions observed in flow-stop experiments. This is due to the higher rotational diffusion at elevated pH levels and in the presence of Helux, leading to a faster decay in alignment. Thus, the SAXS data confirm the observations regarding fibril dynamics from the flow-stop experiments.

The fibril alignment in the double flow-focusing channel actually used for assembly can be simulated numerically via a Smoluchowski equation (see ref 47 for details). Such simulations give a detailed approximation of the alignment state in the channel and can be used to investigate the effect of changes in fibril dynamics. In Figure 9a, the order parameter for the longer fibrils in the dispersion is shown as a function of downstream distance. At each position, the alignment varies over the cross section of the channel, and this variation is indicated in the figure as the shadowed region. The alignment increases but shows large variations after the first sheath. After the second sheath at *x*/*h* = 6, the alignment increases further and the variation decreases drastically, i.e., the alignment is homogeneous over the cross section.

**Figure 9:**
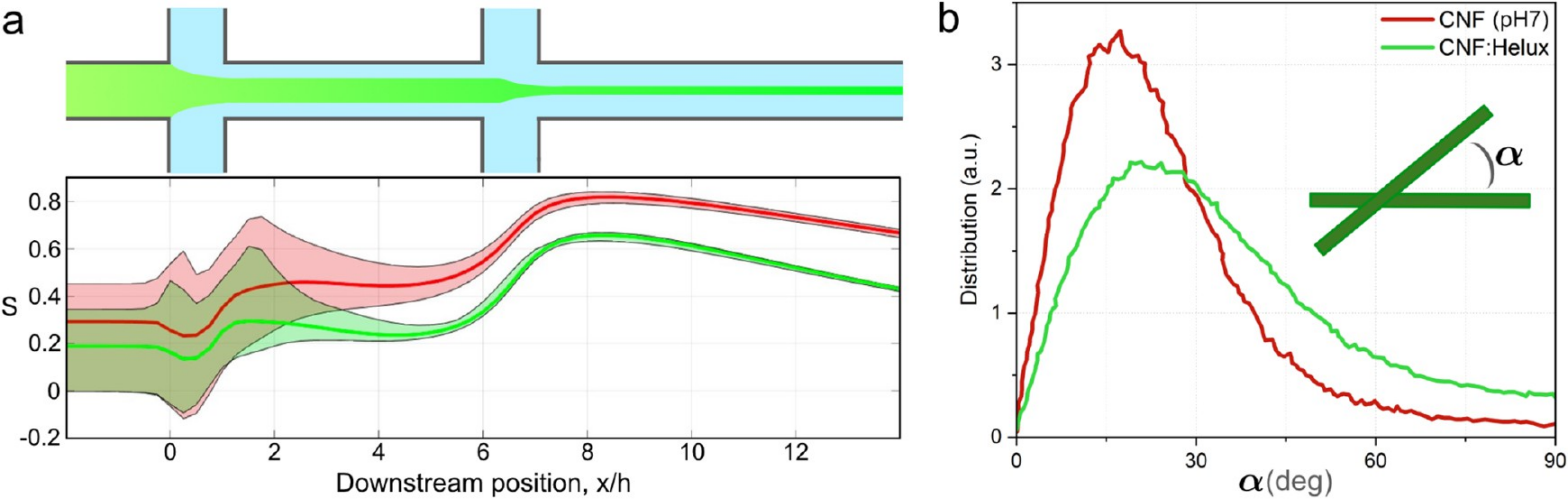
Alignment of the long fibril fraction from the digital twin of fibril alignment during assembly.^47^ The reference pH 7 is shown in red, and a case with a 25% reduction in *D*_r_ (corresponding to CNF:Helux) is shown in green. (a) Estimated mean, maximum, and minimum (over the assembly channel cross section) of the order parameter. (b) Distribution of the angle between fibrils (α) at *x*/*h* = 8.

As an attempt to model the difference between pH 7 (red) and CNF:Helux (green) with the digital twin, pH 7 (green) is modeled as the previously calibrated pure CNF dispersion.^47^ The reduced hydrodynamic length of the long fibrils observed in Figure 7b indicates a reduction of *D*_r_ around 25%, and this reduced *D*_r_ is used for the green curve, showing a reduced maximum alignment.

Furthermore, Figure 9b shows the resulting change in distribution of the relative orientation between neighboring fibrils, and it is seen that the addition of Helux results in not only less aligned fibrils but also that there is a considerably larger angle between them, a factor that affects the nanostructure and fibril/fibril interactions and thus contributes to the differences in mechanical properties (Figure 3).

### Functional Group Assessment and Postfunctionalization of Composite Filaments

The functional group assessment was performed to quantify the accessibility of amine groups in Helux within the composite filaments. Using the ninhydrin assay, we determined the availability of these functional groups for postfunctionalization, which is crucial for understanding how Helux influences the filaments’ properties. The reddish color observed in the CNF:Helux filaments (Figure 3g) indicates the accessibility of some amine groups. Results, summarized in Table 2, show that approximately 7% of the amine groups are accessible. Assuming an even distribution of Helux across the filament cross section, 7% of the cross-sectional area corresponds to a region with a radius of 0.4 μm for a filament with a diameter of 8.5 μm, representing a depth of several nanofiber diameters (∼2.73 nm, as shown in Figure S1d). This depth was determined based on the equilibrium distribution of functional groups, making it a thermodynamic result rather than a kinetic one. This assessment provides insight into the potential for further functionalization and the interaction between Helux and CNF in the filament structure.

**Table 2:** Quantification of Amines in CNF:Helux Filaments (60 mm, Figure S4) and a Solution of Helux 3 μg mL^–1^ Using the Ninhydrin Test

	amount of amines	accessible amine groups
sample	(μmoles)	(%)
Helux (3 μg)	0.161	
composite filament 1	0.011	6.8
composite filament 2	0.010	6.2
composite filament 3	0.012	7.4

## Conclusions

Functional composite filaments can be fabricated by combining the dendritic polyampholyte, Helux, and TEMPO-oxidized CNF using a flow-focusing assembly technique. The results indicate the concentrations and pH conditions at which the composite dispersions are stable, homogeneous, flowable, and spinnable. In short, dispersion pH must be increased, and gelling agent pH must decrease when Helux is added. While factors such as salinity and temperature may also influence interfibrillar interactions, they were not explored here. Future studies could investigate their interplay with pH to further understand Helux-mediated interactions.

Addition of a small amount of Helux to CNF (5:100) dispersion resulted in an elevated strength (about 60%) and a drastic enhancement of toughness (about 5 times) for the composite filaments. According to the WAXS results, composite filaments exhibited a slightly lower degree of alignment (*f*_c_ = 0.8) compared to pure CNF filaments (*f*_c_ = 0.87), resulting in a lower elastic modulus (25%) for the non-cross-linked composite filaments. The SAXS results together with POM flow-stop analyses revealed faster dynamics within CNF:Helux dispersions, indicating that CNFs in composite dispersions exhibit slightly faster decay toward isotropy compared to pure CNF dispersions. The accelerated rotation implies higher fibril mobility and rapid dynamics at elevated pH, influenced by various factors such as the multivalency of Helux, dispersion ionic strength, and pH.

Although composite filaments are less aligned than pure CNF filaments, their ultimate strength and toughness exceed pure CNF filaments due to enhanced interactions facilitated by Helux multivalency and a denser structure exhibited in SEM analyses. Thus, the Helux multivalency fosters stronger bonding interactions within CNFs, contributing to heightened strength and toughness despite reduced alignment. Additionally, the denser structure observed via SEM suggests a more compact arrangement of components within the composite filaments, which likely reinforces its mechanical properties.

Moreover, Helux imparts functionality to the filament, highlighting its role in enhancing both the structural integrity and functional performance. The reddish color of the CNF filament indicates that approximately 7% of Helux’s amine groups are accessible for functionalization, with an accessible layer depth of about 0.4 μm (equal to several nanofiber diameters). While this study focuses on Helux-mediated CNF interactions, polyampholyte properties, such as molecular weight, charge density, and branching, could influence fibril bridging, ionic interactions, and network density. Exploring these factors may help optimize the mechanical performance in future studies.

These findings highlight the trade-off between alignment and mechanical properties, demonstrating that while alignment enhances stiffness, strong interfibrillar interactions facilitated by Helux can compensate for the reduced alignment, leading to superior strength and toughness. This challenges the traditional notion that high alignment is the sole driver of performance in CNF-based materials. Future studies may further optimize this balance by tuning the molecular properties of the Helux, providing insights for designing next-generation functional cellulose-based materials.

## Supplementary Material



## Notes

The following files are available free of charge: The Supporting Information is available free of charge at https://pubs.acs.org/doi/10.1021/acs.biomac.5c00128.

AFM images of CNF (pH 7) and composite dispersion together with the distribution of length and diameter of CNFs; apparent hydrodynamic length distribution based on POM flow-stop results; Helux structure; illustration of the flow-focusing setup; flow sweep measurements of CNF (pH 7 and pH 10) and CNF:Helux dispersions; calibration curve of N-butylamine solutions at absorbance at λ = 570 nm; and disperse red succinic *N*-hydroxysuccinimide (DredS-NHS) structure (PDF)
