# Supplementary material for: Tuning Alignment, Strength, and Toughness in Functional Cellulose:Helux Filaments: A Molecular Trade-Off
Source: Biomacromolecules. 2025 Jun 28;26(7):4133–45. doi: 10.1021/acs.biomac.5c00128 (PMC12264946; doi:10.1021/acs.biomac.5c00128)
Supplement: Supplementary file 1 [file bm5c00128_si_001.pdf]

Supporting Information
Tuning Alignment, Strength, and Toughness in Functional Cellulose:Helux Filaments: A Molecular Trade-off

Saeed Davoodi,[†,‡] Faridah Namata,[¶,‡] Tomas Rosén,[¶,‡] Stephan V. Roth,[§,¶,‡] Michael Malkoch,[¶,‡] L. Daniel Söderberg,[¶,‡] and Fredrik Lundell[*,†,‡]

†*Department of Engineering Mechanics, KTH Royal Institute of Technology, 100 44 Stockholm, Sweden*

‡*Wallenberg Wood Science Center, KTH Royal Institute of Technology, 100 44 Stockholm, Sweden*

¶*Department of Fibre and Polymer Technology, KTH Royal Institute of Technology, 100 44 Stockholm, Sweden*

§*Deutsche Elektronen-Synchrotron DESY, D-22607 Hamburg, Germany*

E-mail: frlu@kth.se

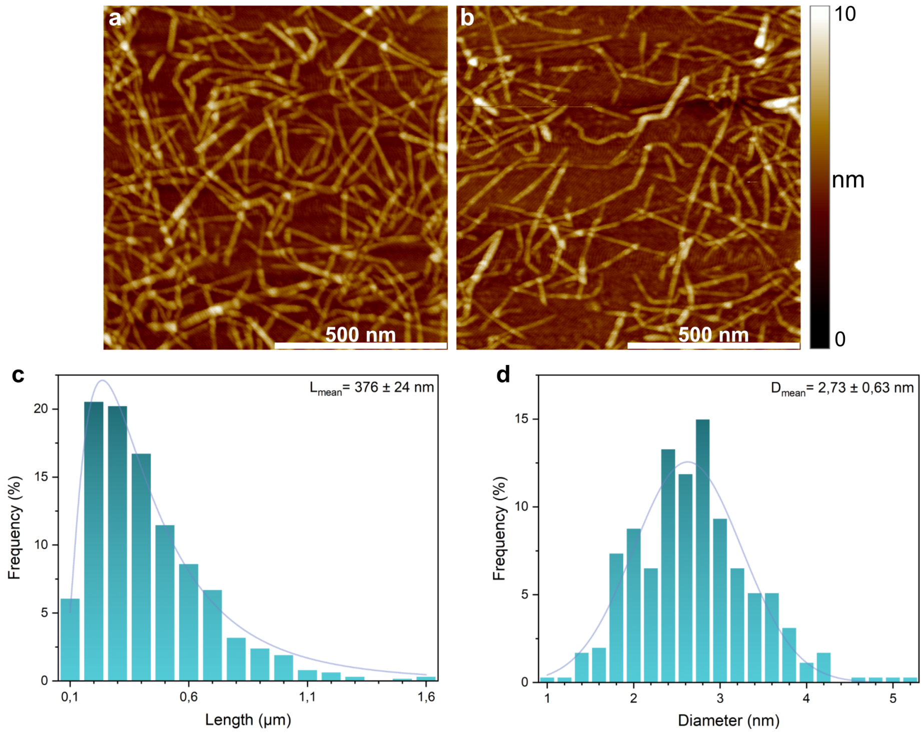

Figure S1: AFM images of a) CNF (pH 7) and b) CNF:Helux 100:5 dispersions proving no aggregation after addition of Helux. Distribution of c) length and d) diameter of the CNFs.

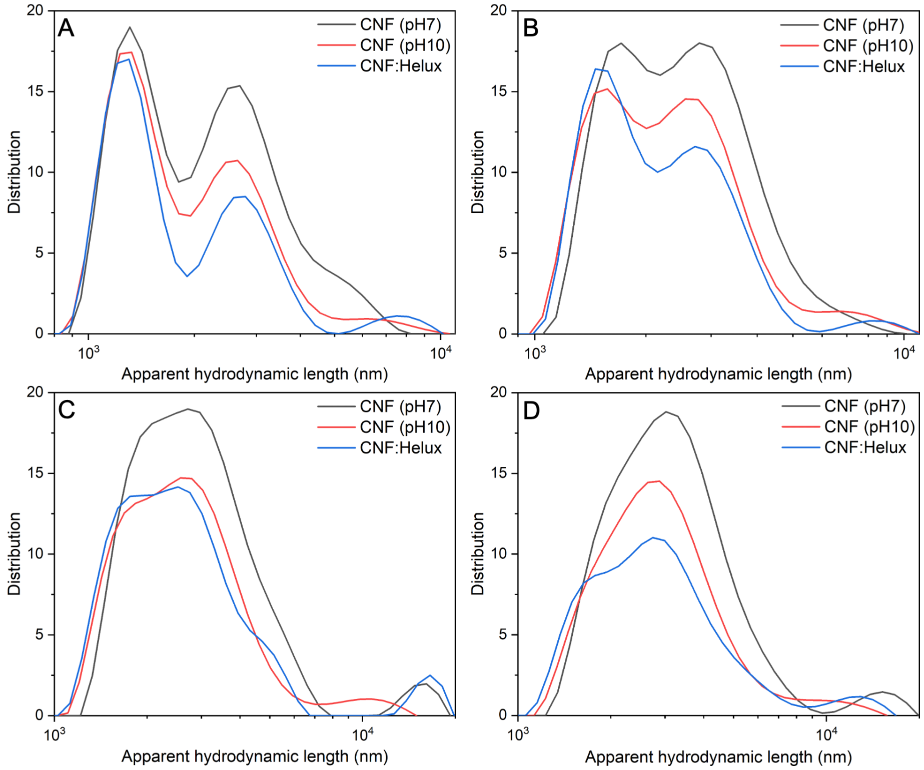

Figure S2: Apparent hydrodynamic length distribution at different positions (based on Figure 5 of flow focusing channel for CNF (pH 7), CNF (pH 10) and CNF:Helux.

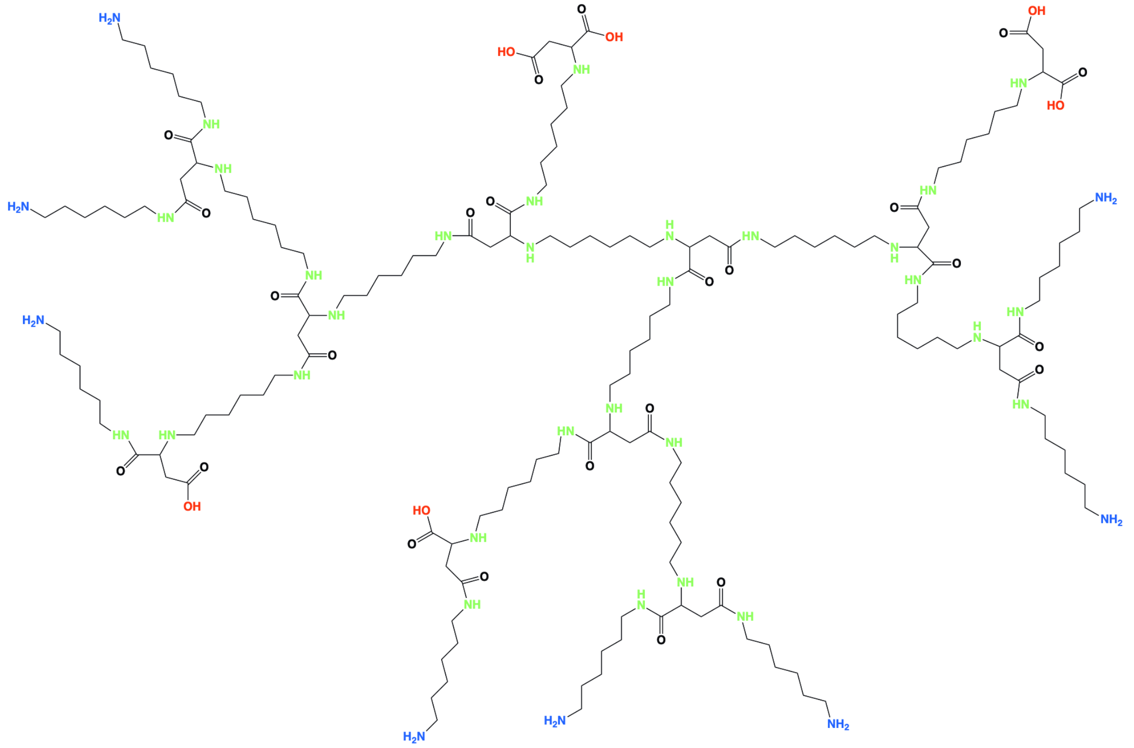

Figure S3: Helux structure.[1]

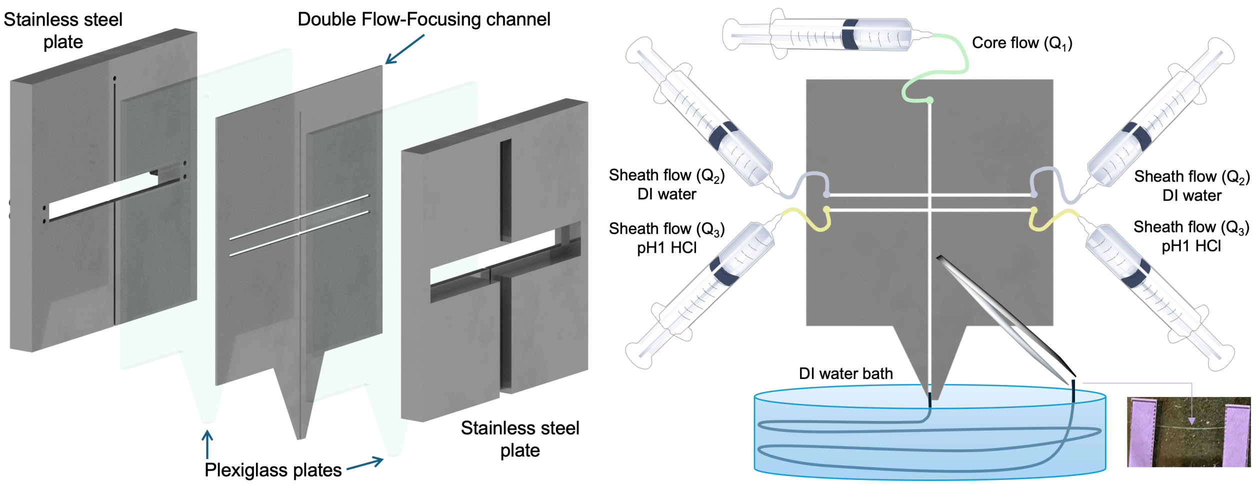

Figure S4: Illustration of the flow-focusing setup used to assemble composite filaments. Channels in the flow-focusing geometry were all 1 mm wide.

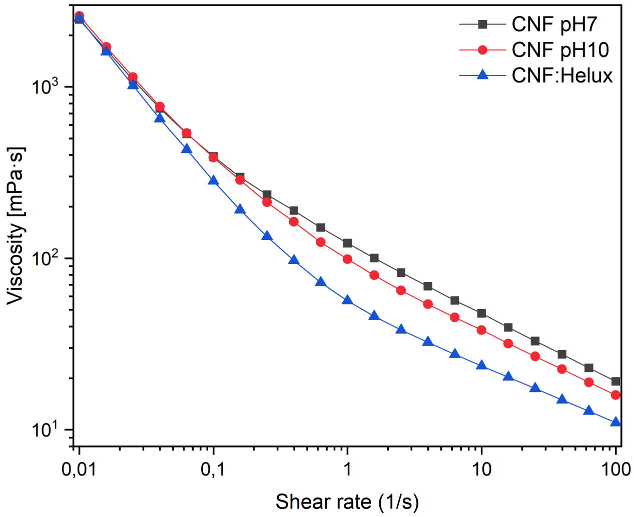

Figure S5: Flow sweep measurements of CNF (pH 7 and pH 10) and composite dispersions.

Helux quantification

Estimated amount of Helux in a filament was obtained as follows. Assuming 1μg mm^{-1} is the weight of a filament containing 5% Helux, it contains 0.05 μg mm^{-1} Helux. A metal pin contains on average 60 mm of filament (Figure S6) with 3 μg Helux.

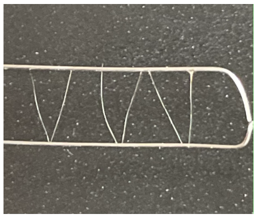

Figure S6: 60 mm of CNF:Helux filament attached on a metal pin.

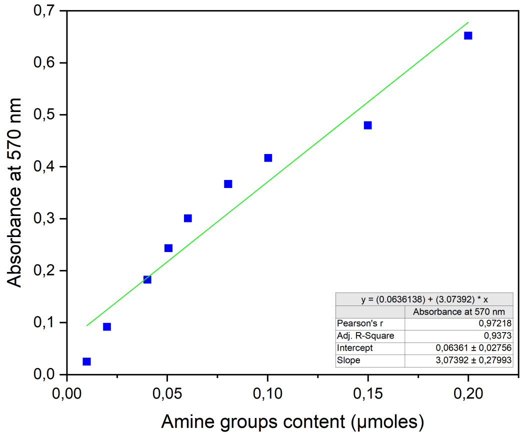

Figure S7: Calibration curve of N-butylamine solutions at absorbance at $\lambda = 570\ nm$.

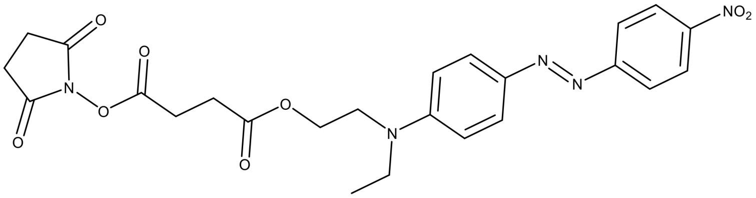

Figure S8: Disperse red succinic N-hydroxysuccinimide (DredS-NHS) structure.[2]

References

(1) Ingverud, T.; Malkoch, M. Helux: a heterofunctional hyperbranched poly (amido amine) carboxylate. *ACS Appl. Polym. Mater.* **2019**, *1*, 1845–1853.

(2) Ingverud, T.; Erlandsson, J.; Wågberg, L.; Malkoch, M. Dendritic Polyampholyte-Assisted Formation of Functional Cellulose Nanofibril Materials. *Biomacromolecules* **2020**, *21*, 2856–2863.
